# Role of ursolic acid in preventing gastrointestinal cancer: recent trends and future perspectives

**DOI:** 10.3389/fphar.2024.1405497

**Published:** 2024-07-24

**Authors:** Abhishek Chauhan, Vinay Mohan Pathak, Monika Yadav, Ritu Chauhan, Neelesh Babu, Manish Chowdhary, Anuj Ranjan, Darin Mansor Mathkor, Shafiul Haque, Hardeep Singh Tuli, Seema Ramniwas, Vikas Yadav

**Affiliations:** ^1^ Amity Institute of Environmental Toxicology Safety and Management, Amity University, Noida, Uttar Pradesh, India; ^2^ Parwatiya Shiksha Sabha (PASS), Haldwani, India; ^3^ Cancer Biology Laboratory, School of Life Sciences, Jawaharlal Nehru University, New Delhi, India; ^4^ Department of Biotechnology, Graphic Era Deemed to be University, Dehradun, Uttarakhand, India; ^5^ Department of Microbiology, Baba Farid Institute of Technology, Dehradun, Uttarakhand, India; ^6^ CSIR-Institute of Genomics and Integrative Biology, New Delhi, India; ^7^ Academy of Biology and Biotechnology, Southern Federal University, Rostov-on-Don, Russia; ^8^ Research and Scientific Studies Unit, College of Nursing and Allied Health Sciences, Jazan University, Jazan, Saudi Arabia; ^9^ Gilbert and Rose-Marie Chagoury School of Medicine, Lebanese American University, Beirut, Lebanon; ^10^ Department of Bio-Sciences and Technology, Maharishi Markandeshwar Engineering College, Maharishi Markandeshwar (Deemed to Be University), Ambala, India; ^11^ University Centre for Research and Development, University Institute of Pharmaceutical Sciences, Chandigarh University, Mohali, India; ^12^ Department of Translational Medicine, Clinical Research Centre, Skåne University Hospital, Lund University, Malmö, Sweden

**Keywords:** ursolic acid, gastrointestinal cancer, nanoformulations, anticancer therapy, molecular profiling, nanoparticle-based drug delivery

## Abstract

Gastrointestinal malignancies are one of the major worldwide health concerns. In the present review, we have assessed the plausible therapeutic implication of Ursolic Acid (UA) against gastrointestinal cancer. By modulating several signaling pathways critical in cancer development, UA could offer anti-inflammatory, anti-proliferative, and anti-metastatic properties. However, being of low oral bioavailability and poor permeability, its clinical value is restricted. To deliver and protect the drug, liposomes and polymer micelles are two UA nanoformulations that can effectively increase medicine stability. The use of UA for treating cancers is safe and appropriate with low toxicity characteristics and a predictable pharmacokinetic profile. Although the bioavailability of UA is limited, its nanoformulations could emerge as an alternative to enhance its efficacy in treating GI cancers. Further optimization and validation in the clinical trials are necessary. The combination of molecular profiling with nanoparticle-based drug delivery technologies holds the potential for bringing UA to maximum efficacy, looking for good prospects with GI cancer treatment.

## 1 Introduction

**FIGURE 1:**
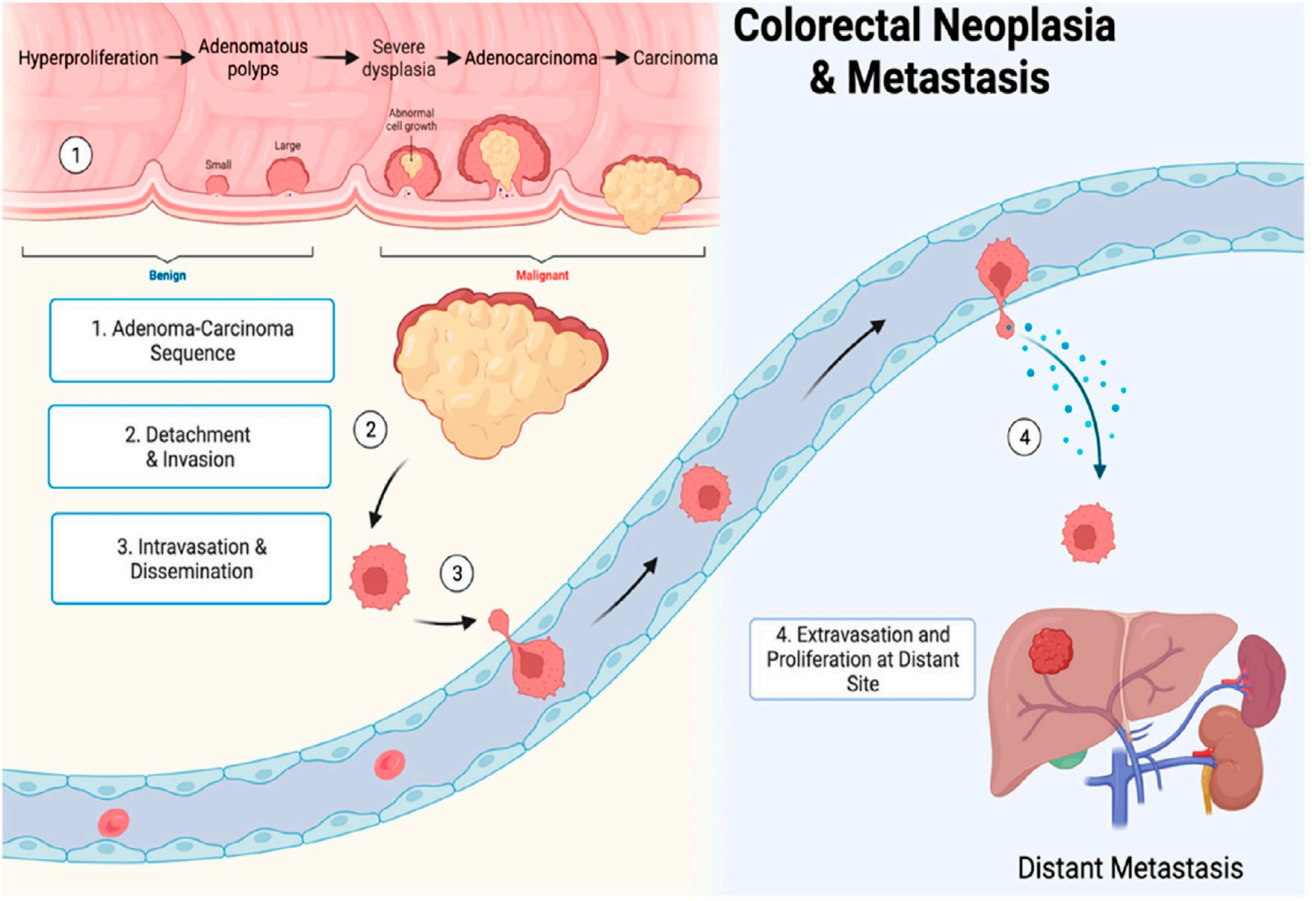
Gastrointestinal cancer formation and metastasis [adopted from Seely et al. (2022)].

The term gastrointestinal cancer refers to a heterogeneous group of cancers with varying origins and expressions. It could be genetic and has the potential to spread throughout the entire gastrointestinal tract, from the esophagus to the rectum, including vital organs like the liver. Among the globally reported cancers gastrointestinal cancer accounts for one-fourth of the prevalence (Arnold et al., 2020; Sung et al., 2021). As per the research published by Wang et al. (2024), the data collected from 185 countries showed the prevalence of stomach, liver, oesophagus and gall bladder among East Asian countries. While Western European countries are more prone to pancreatic cancer, New Zealand has a high incidence rate of colorectal cancer. The formation and metastasis of GI cancer are shown in Figure 1. As far as the treatment for gastrointestinal cancer is concerned, surgery, chemotherapy and radiotherapy have been standard practices for decades. Moreover, it may reoccur even after the surgery, and detection rate remains low posing a limitation for such practices. Additional strategies such as immunotherapy, particle therapy, photodynamic therapy, targeted therapy, and combination therapies are progressively gaining attraction (Rawla and Barsouk, 2019; Fan et al., 2022).

**FIGURE 2:**
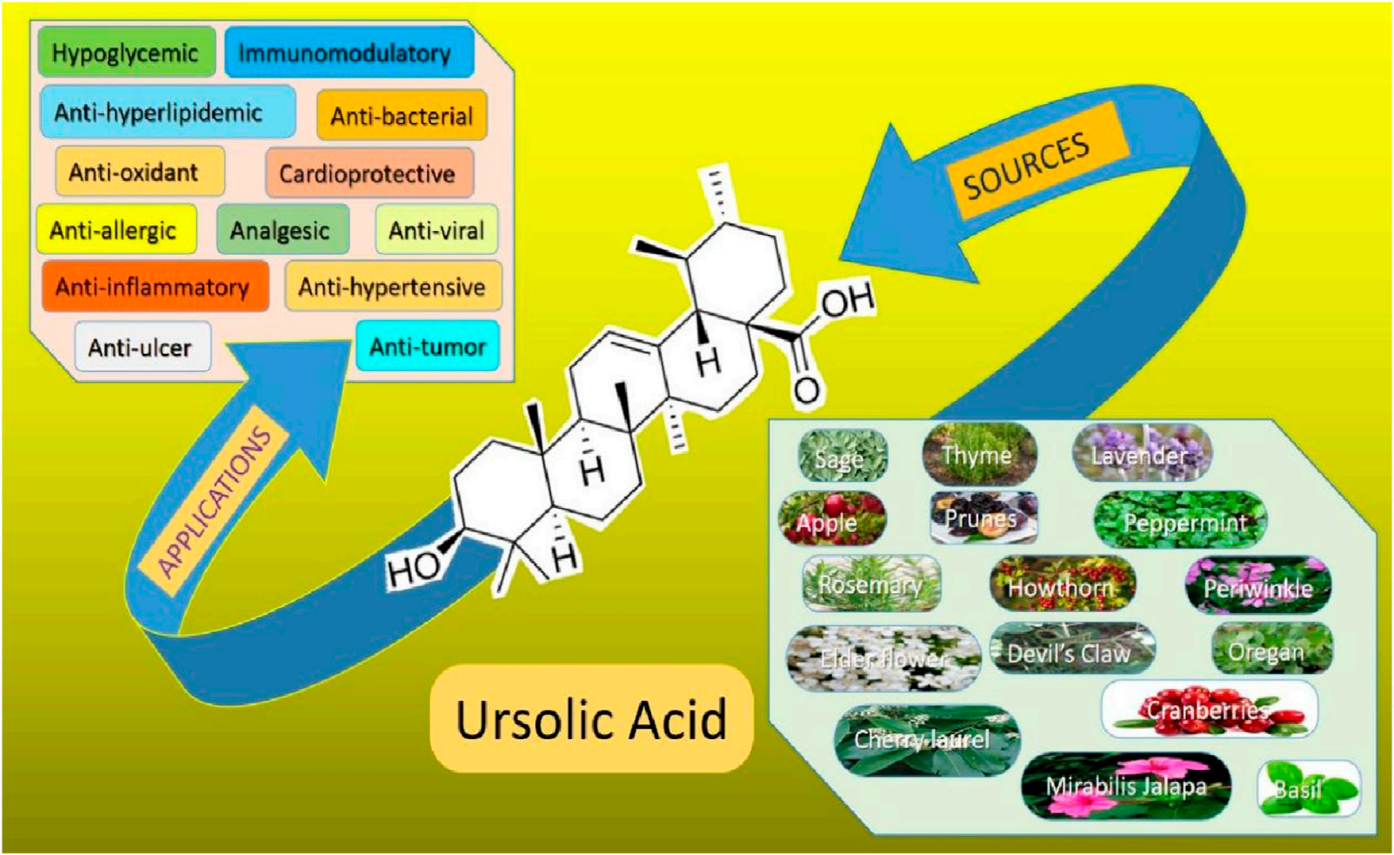
Ursolic acid sources and its application in different infection and disease treatments.

Globally, stomach cancer in adolescents and young adults (AYA) caused 49,000 incident cases, 27,895 deaths, and 1.57 million DALYs (disability-adjusted life years) in 2019 (Zhang et al., 2023). In 2020, global lifetime risks of developing gastrointestinal cancers were 8.20% from birth to death while the mortality risk was 6.17%. The highest risk was for colorectal cancer having a total lifetime risk of 38.5% and a mortality rate of 28.2% from all gastrointestinal cancers followed by stomach, liver, esophagus, pancreatic, and gallbladder cancers (Wang S. et al., 2024). Consequently, effective treatment with fewer adverse effects which is economical, and readily available is urgently needed. UA is one of the active substances widely reported for its antidiabetic, anti-inflammatory and antioxidant properties as shown in Figure 2 (González-Garibay et al., 2020; Wang et al., 2020; Somantri et al., 2021). UA is found in a wide variety of fruits, vegetables and herbs such as *Hedyotisdiffusa* sp., *Gargenia* sp. (Woźniak, et al., 2015; Alam et al., 2021). UA is also being recognized for its broad spectrum anticancer properties. Additionally, it is well known for its anti-diabetic, anti-inflammatory and antioxidant properties (Kim et al., 2018; Mu et al., 2018; Yin et al., 2018; Lin et al., 2019; Lin et al., 2019; Kang et al., 2021a; Wang et al., 2021).

**FIGURE 3:**
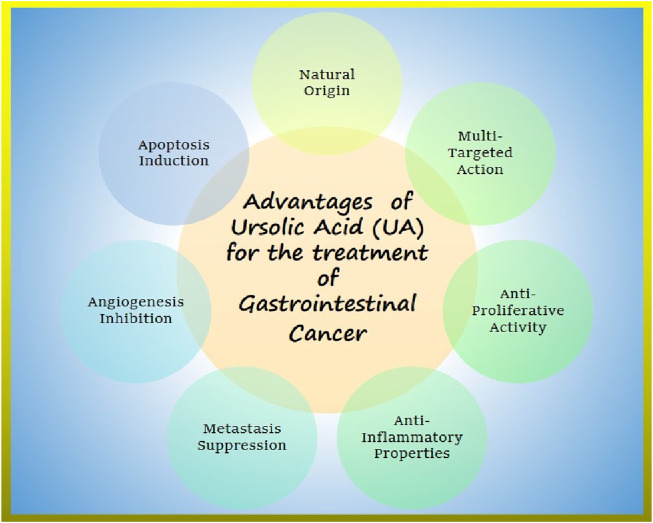
Ursolic acid advantages in gastrointestinal cancer treatment.

Research supports the potential of UA in promoting apoptosis, limiting angiogenesis, and overcoming therapeutic resistance, thereby advancing the treatment of GI cancer as shown in Figure 3 (Wang et al., 2016; Guo et al., 2019; Kang et al., 2021a; Akshit et al., 2023). Moreover, UA has been demonstrated to enhance chemosensitivity of gemcitabine in pancreatic cancer cases as it induces apoptosis and autophagy (Lin et al., 2020). Shanmugam et al. (2011) reported that UA can suppress the spread of prostate cancer to the lungs and liver by deactivating the C-X-X motif chemokine receptor 4(CXCR4) in TRAMP mice.

The anticancer activities of the UA are due to its ability to regulate the tumor microenvironment (Shanmugam et al., 2011; Zhang N. et al., 2020; Zhang X. et al., 2020). UA holds a significant position among many triterpenoids due to its wide range of biological activities (Panda et al., 2022).

## 2 Chemistry and pharmacokinetics

### Chemistry

**FIGURE 4:**
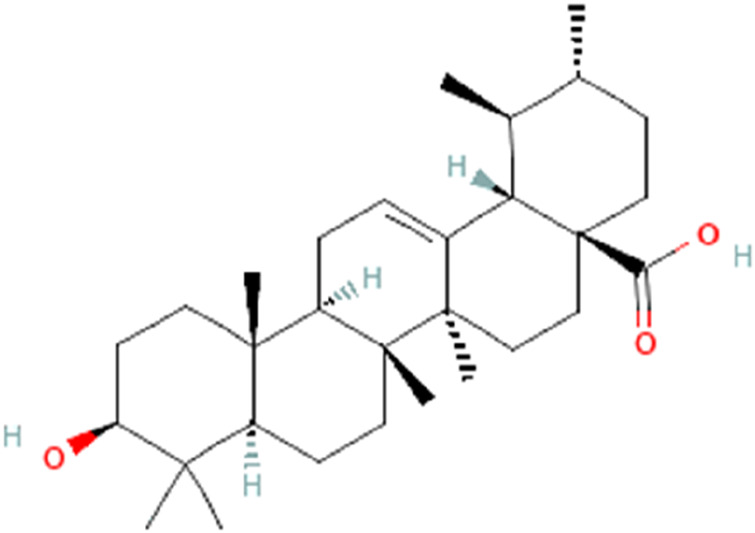
Chemical structure of ursolic acid.

UA is widely present in various plant sources, including medicinal plants, fruits, herbs, etc., as a pentacyclic triterpenoid with a characteristic pentacyclic structure and hydrophobic tail, defining its unique features. It is chemically represented by the formula C_30_H_48_O_3_ (Figure 4) (Ikeda et al., 2008). Its stability and capacity to interact with different biological processes in the body are due to this structure (Liu et al., 2021). The lipophilic character of UA contributes towards its oral bioavailability. The chemical structure of the substance is crucial because of its interaction with the cell membrane which allows the gastrointestinal tract to absorb it (Alam et al., 2021). Due to its potential anti-cancer activity, UA has gained lots of attention, especially in the context of gastrointestinal tumors (Pięt and Paduch, 2019; Limami et al., 2023). The structure-activity relationship for terpenoids and their derivatives has been described at the molecular level by the development of a 3D-QSAR model (Perestrelo et al., 2019). The development of machine learning algorithms has facilitated explorations in this area. A comparative molecular field analysis (CoMFA) model, for example, displays an excellent cross-validation correlation coefficient (q2) of 0.54 and a regression correlation coefficient (r2) of 0.86 (Stitou et al., 2019). Since T9 and B42 exhibit good binding affinities and fall within the conventional limits of all filters, they have been designated as the greatest hits (Yadav et al., 2018). The cytotoxic activity of UA analogs against human lung (A-549) and CNS (SF-295) cancer cell lines was predicted using QSAR models. The regression coefficient (r2) and cross-validation regression coefficient (rCV2) of the QSAR model were found to be 0.85 and 0.80, respectively, for cytotoxic activity against the human lung cancer cell line (A-549) (Kalani et al., 2012).

### Pharmacokinetics

Orally bioavailable, UA is mostly absorbed in the gastrointestinal tract. Since UA is lipophilic, it can pass through cell membranes and be absorbed more easily (López-Hortas et al., 2018; Mlala et al., 2019). After absorption, UA is disseminated throughout the body, even to regions impacted by gastrointestinal malignancies. Tissue permeability and protein binding are two variables that could affect its distribution (Mlala et al., 2019; Wang et al., 2021). The conversion of UA involves hepatic metabolism. Water-soluble metabolites are formed in part by phase II conjugation processes such as glucuronidation and sulfation. Biliary excretion is the main mechanism by which UA and its metabolites are removed from the body. Prolonged UA presence in the system could be caused by enterohepatic circulation (Yu et al., 2020).

## 3 Major gastrointestinal (GI) cancer

Gastrointestinal cancers, are the most prevalent types of cancer across the globe. They arises due to the uncontrolled cell growth in the organs of gastrointestinal tract. Moreover, if they are not detected early, they tend to be lethal for the affected individuals. Considering the risk factors associated with this type of cancer, it is essential to manage effective prevention strategies (Souza et al., 2024). Fortunately, UA is naturally present in various herbs and fruits such as peels of apples, basil, etc., which can serve as functional foods. Several studies on its pharmacological properties have suggested its broad-spectrum potential in inhibiting the initiation and progression of various types of GI cancers (Table 1). It inhibits cell proliferation by triggering cell apoptosis in several studied clinical models. Additionally, its anti-inflammatory properties enable it to address inflammation associated with GI cancers. UA also possess antioxidant properties by which it actively counters carcinogen-associated oxidative stress. It can actively inhibit advanced cancer stages via the suppression of epithelial-mesenchymal transitions (EMT) and the expression of matrix metalloproteinase (Zhao M. et al., 2023). The synergistic effects of UA with several other conventional chemotherapeutic agents were also studied. Optimization of its formulation and delivery methods, including novel drug delivery systems like nanoparticles (NPs) and liposomes, holds promise for enhancing its bioavailability and therapeutic potential. As research advances, UA stands as a compelling natural compound with significant chemo-preventive properties against GI cancer (Kadasah and Radwan, 2023).

**TABLE 1:** Brief overview on the effect of UA on cancers of gastrointestinal origin.

S. No	Type of cancer	Subjective model	Physiological effects	Mechanism of action	References
1	Colon cancer	HT-29	Induces apoptosis	↓ EGFR/MAPK, ↓ Bcl-2/Bcl-xL, ↑ caspase-3/caspase-9	Shan et al. (2009)
2	Colorectal cancer	CRC SW620	Inhibits proliferation, migration and clonality, Induces cell cycle arrest	↓ Wnt/β-catenin signaling	Zhao H. et al. (2023)
3	Esophageal cancer	TE-8 and TE-12 ESCC cells	Inhibits proliferation and viability	Induces autophagy, ↑ LC3-II, ↓ p62, ↓ AKT	Lee et al. (2020), Meng et al. (2021)
4	Gallbladder cancer	GBC-SD and SGC996	Inhibits proliferation, Induces apoptosis and cell cycle arrest	↑ caspase-3, ↑ caspase-9, ↑ PARP, ↑ Bax, ↓ Bcl-2	Weng et al. (2014)
5	Gastric cancer	SNU-484	Inhibits proliferation and invasion, Induces apoptosis	↑ caspase-3, ↑ caspase-9, ↑ PARP, ↑ Bax, ↓ Bcl-2, ↑phospho-P38, ↑ phospho-JNK, ↓ MMP-2	Kim and Moon (2015), Xiang et al. (2015)
6	Hepatocellular carcinoma	HepG2, Hep3B, Huh7, SSMC-7721	Inhibits proliferation, migration, invasion, colony formation	↓ STAT3, ↓ JAK2, ↓ phospho-AKT, ↓ Bcl2, ↑ phospho-ERK	Liu et al. (2017)
7	Intestinal cancers	INT-407 and HCT-116	Inhibits proliferation and migration, Induces apoptosis	↑ Apoptotic genes (BAX, P21, P53), ↓ Survival factor genes (Bcl2, Survivin, SP1, NFκB), ↓ migration genes (FN1, CDH2, CTNNB1, TWIST)	Rawat and Nayak (2021)
8	Pancreatic cancer	MIA PaCa-2, PANC-1 and Capan-1	Inhibits proliferation and viability, Induces apoptosis	↑ caspase-3/7, ↑ caspase-8/9, ↑ phospho-JNK, ↓ PI3K/Akt/NF-κB pathways	Li et al. (2012)

### 3.1 Esophageal cancer

Esophageal cancer (cancer in esophagus) serves as a global concern in cancer research and is challenging due to its unpredicted disease pattern. In the current scenario, advanced molecular techniques are needed to increase our understanding of diagnostic, disease prediction and treatment approaches (Lagergren et al., 2017). In addition to challenges associated with diagnostic and treatment practices, there are further challenges in controlling the progressive occurrence of GI cancers. Cancer cases are rapidly increasing throughout the world, with reports emerging from regions such as Africa, Eastern Europe and Eastern Asia. Cancer epidemiology-based studies helps to study the prevalence, incidence, and mortality effects. In the case of esophageal cancer, two types of epidemiological patterns are observed: namely, adenocarcinoma and squamous cell carcinoma. Squamous cell carcinoma of the esophagus is more common compared to adenocarcinoma; with case studies indicating that approximately 90% of cases are squamous cell carcinoma. Esophageal cancer is majorly reported in aged people and rarely reported in the younger generation. Squamous cell carcinoma cases are found in excessive alcoholic and tobacco consumption persons while adenocarcinoma is related to gastroesophageal effects like obesity (Rustgi and EI-Serag, 2014). Reports based on molecular studies found mutations play an important role in the progressive development of esophageal cancer. Such mutations cause adverse changes in the signaling pathways of the vital gene and have a role in tumour supersession. CDKN2A genes encode tumor suppressor protein p16 and mutations in such gene cause gene alterations in CDKN2A, which is further responsible for disease progression (Secrier et al., 2016; Thrift, 2016). Similar results have been reported for p53 protein-coding genes, i.e., TP53, in the case of squamous cell carcinoma and adenocarcinoma. The p53 protein has a significant role in tumor suppression and defects in its encoding gene TP53, leading to progressive tumor formation in adenocarcinoma and squamous cell carcinoma both conditions (Song et al., 2014).

Cellular migration, proliferation and survival are controlled by epidermal growth factor receptor (EGFR) pathways. In esophageal adenocarcinoma, EGFR genes are often overexpressed and unregulated. The signaling pathways of EGFR promote metastases, angiogenesis and tumor growth. Therefore, targeting these are one of the approachable ways of cancer treatments (Liu et al., 2017). EGFR signaling pathway associated with PI3K, AKT, mTOR cascade and uncontrolled regulation in these proteins lead to enhanced cellular proliferation, exhibits resistance to treatment therapy, and develop cancer in the body (Xie et al., 2020). Esophageal adenocarcinoma initiation is linked with the Wnt/β-catenin pathway. WNT signaling pathway dysregulation promotes the excessive growth of tumor cells, and the aberrant activation of β-catenin facilitates metastasis by translocating to the nucleus. Targeting the Wnt/β-catenin signaling for treatment is considered a promising therapeutic approach for esophageal cancer (Hassanabad et al., 2020). It has been reported that Notch signaling plays a role in esophageal cancer as it assists in the tumor formation. In a preclinical study, inhibitors of the Notch signaling pathway have demonstrated anti-tumor activity against esophageal cancer. Therefore, targeting the Notch signaling pathway is considered a promising therapy for esophageal cancer (Wang et al., 2014).

Exploring the application of molecular biomarkers in the diagnosis and early detection of esophageal cancer holds promise as an effective approach towards cancer management. The altered DNA methylation patterns, microRNA expression, and ctDNA have emerged as crucial biomarkers for assessing risk factors, early detection, and monitoring of treatment response. Additionally, molecular subtyping may also support in identifying patients who are prone to benefit from specific treatments, such as immunotherapy and neoadjuvant chemotherapy (Shapiro et al., 2015). Although much progress has been made, barriers exist that make it difficult to translate molecular data for clinical use. Heterogeneity within the tumors of the esophagus, tumor microenvironment dynamics, and interpatient variability warrants the need for an extensive view of the molecular landscape that can be used for rational treatment decisions. Integration of molecular profiling into everyday clinical practice needs to have standardized methodologies, robust biomarkers, and collaborative research efforts to validate findings and ascertain clinical utility. Molecular research reoriented has reshaped our understanding of esophageal cancer, being the source of understanding its mechanisms, classification, and treatment options. Through clarifying key molecular abnormalities, researchers have outlined early detection biomarkers, prognostication biomarkers, and biomarkers for targeted therapy selection. Recent progress in molecular characterization has deeply refined esophageal cancer, allowing more focused diagnoses and individualized treatment strategies. Molecular subtypes defined by gene expression profiles are distinct and have specific biological features and outcomes (Sheikh et al., 2023).

### 3.2 Gastric (stomach) cancer

*Helicobacter pylori* is a bacterium associated with the stomach lining and it is one of the risk factors associated with gastrointestinal cancer. It causes chronic gastritis and peptic ulcers which leads to cancer of the stomach by creating an environment for the carcinogens (Chen et al., 2024). Furthermore, dietary habits such as smoking, and consuming salty food, tobacco, and alcohol consumption increase the potential risk for gastric cancer.

The location of the tumor and the various stages of gastric cancer influence its clinical representations. Several non-specific symptoms such as nausea, loss of appetite, bloating, abdominal discomfort, etc., May count under the early symptoms of gastric cancer. With the progression of cancer, more pronounced symptoms may appear such as persistent vomiting, weight loss, fatigue, anemia, etc. It is very important to recognize these early signs for better treatment (Shin and Park, 2024). For the diagnosis of such conditions, there are several approaches available in the clinical settings from imaging to endoscopy following biopsy. The direct visualization of the stomach lining can be achieved using endoscopy for the appropriate samples for biopsy. Several advanced techniques for imaging including endoscopic ultrasound, computed tomography, magnetic resonance imaging, etc., Allow the detection of the extent of tumor invasion and its metastasis to the adjacent tissues and different organs (Huang et al., 2023). The samples which are taken for the biopsy may be subjected to molecular characterization for the identification of genetic alterations, treatment, and prognosis. Overall strategies for the treatment depend on the patient’s stage of cancer and current health condition whether it is required to remove tissue surgically or following treatment with chemotherapy (Alsina et al., 2023). Usually, for the treatment of gastric cancer, chemotherapy is commonly employed using drugs such as fluoropyrimidines, platinum-based agents and taxanes. The targeted therapies are employed for molecular pathways which are actively associated with the progression of tumor. Trastuzumab is one of the best examples, it is a monoclonal antibody which specifically targets the HER2/neu receptor involved in gastric cancer (Hui et al., 2024).

As far as immunotherapy is concerned, immune checkpoint inhibitors that particularly target protein PD-1 or its ligand PD-L1 (programmed cell death protein) are widely employed due to their promising results (Zou et al., 2019; Zou et al., 2024). Moreover, current studies suggest that UA possesses different pharmacological properties that can inhibit gastric cancer development.

#### 3.2.1 Anti-proliferative effects

UA has demonstrated significant anti-proliferative activity against gastric cancer cells in preclinical studies. By inhibiting cell cycle progression and promoting apoptosis, UA effectively suppresses the uncontrolled growth of cancer cells within the stomach lining (Zhang et al., 2024).

Chronic inflammation plays a crucial role in gastric carcinogenesis, and UA has been shown to exert potent anti-inflammatory effects. By modulating inflammatory signaling pathways and reducing the production of pro-inflammatory mediators, UA may help mitigate the inflammatory microenvironment within the stomach, thereby inhibiting tumor initiation and progression. UA has been found to inhibit angiogenesis (new blood vessel formation important for essential for metastasis and tumor growth) by targeting key angiogenic factors and signaling pathways, thereby depriving tumors of the nutrients and oxygen needed for their sustained growth. Metastasis is a major hallmark of advanced gastric cancer and is associated with poor prognosis. UA has been shown to inhibit the invasion and migration of gastric cancer cells, as well as the formation of metastatic colonies, through its modulation of EMT and metastasis-related signaling pathways (Zou et al., 2019).

In addition to its therapeutic effects, UA may also possess chemo-preventive properties against gastric cancer. Epidemiological studies have suggested an inverse association between dietary intake of UA-rich foods and the risk of gastric cancer development, highlighting the potential preventive role of this natural compound. UA may enhance the efficacy of conventional chemotherapy agents when used in combination. Preclinical studies have shown synergistic interactions between UA and various chemotherapeutic drugs, resulting in enhanced cytotoxicity and apoptosis induction in gastric cancer cells.

### 3.3 Colorectal cancer

Colorectal cancer (CRC) is also the most frequent and second most lethal cancer in the world. It arises from the colonic mucosal epithelia lining causing irregular proliferation of colonocytes (Sachdeo et al., 2020; Sung et al., 2021; Xi and Xu, 2021). In the calendar year 2020, around 1.9 million new cases and 930,000 deaths were reported with the projection of 3.2 million novel cases per year followed by 1.6 million deaths. This report showed an increment of around 60% in new cases and 73% in deaths up to the end of 2040 (Biller and Schrag, 2021; Sawicki et al., 2021). Preclinical studies have revealed the promising efficacy of UA against CRC by inhibiting its proliferation and induction of apoptosis (Chen et al., 2022). Due to its anti-inflammatory properties, UA inhibits the expression of enzymes and cytokines responsible for the inflammation in CRC. Additionally, it contributes to the reduction of oxidative stress through its antioxidant activities, which play a role in CRC development. Antioxidant activities of UA such as free radical scavenging activity reduce the oxidative damage to the DNA (Lin et al., 2013; Zhao M. et al., 2023). Zhang and colleagues demonstrated that UA prevents the growth of malignant cells by modulating the miR-140-5p (increasing)/TGF- β3 (decreasing) axis, which is closely linked to the blockade of the Wnt/β-catenin signaling pathway, potentially inhibiting cell growth (Zhang et al., 2024). Conclusively all the *in vitro* studies concludes that UA can regulate the Wnt/β-catenin, PI3K/Akt, and NF-κB signaling pathway, all of which play significant roles in CRC (Lin et al., 2013; Chan et al., 2019).

### 3.4 Pancreatic cancer

One area of particular interest is the effect of UA on pancreatic cancer, which is known to be highly aggressive and often renders fatal malignancy due to limited treatment options. Several studies have explored the potential of UA in inhibiting the growth and progression of pancreatic cancer cells both *in vitro* and *in vivo* (Prasad et al., 2016; Khwaza et al., 2020; Lin et al., 2020; Lin et al., 2020). UA has been found to exhibit its anticancer effects by inducing apoptosis, and inhibiting migration and invasion (Lin et al., 2020; Hashem et al., 2022). UA has been demonstrated to modulate various signaling pathways implicated in pancreatic cancer development and progression. For instance, it can suppress the activation of NF-κB, a transcription factor involved in inflammation and cancer, thereby reducing the expression of pro-inflammatory and pro-survival genes (Li et al., 2021). Furthermore, UA has been shown to inhibit the PI3K/Akt/mTOR pathway, which is frequently dysregulated in pancreatic cancer, leading to promotion of cell growth and survival (Zafar et al., 2022). In preclinical animal models of pancreatic cancer, UA has exhibited promising antitumor effects, leading to reduced tumor growth and improved survival outcomes (Li et al., 2021). Despite some encouraging findings, further research is needed to fully elucidate the therapeutic potential of UA in pancreatic cancers. Further clinical trials for evaluating the efficacy and safety of UA either alone or in combination with conventional therapies are required (Zafar et al., 2022). Synergistic studies of UA with other phytochemicals to tackle pancreatic cancers could also be evaluated. Also, studies associated with investigating the optimal dosage, formulation, and route of administration of UA are essential for its successful therapeutic development (Wang et al., 2021).

### 3.5 Liver cancer

Studies have shown the anti-cancer properties of UA against hepatocellular carcinoma (HCC), both *in vitro* and *in vivo* (Sureda et al., 2021). UA has been found to exert its effects by multiple signaling pathways including inhibition of STAT3/PD-L1 signaling (Kang et al., 2021b). One of the key physiological aspects is the induction of apoptosis by regulating caspase-3, in liver cancer HepG2 cells and mice models (Ma et al., 2021). By triggering apoptosis, UA can inhibit the uncontrolled growth and proliferation of cancer cells, thereby suppressing tumor progression (Limami et al., 2023). Moreover, UA has been shown to inhibit the migration and invasion of liver cancer cells, which are essential steps in metastasis, and the spread of cancer to other parts of the body (Liang et al., 2021). This anti-metastatic effect is crucial for preventing the aggressive spread of liver cancer and improving patient outcomes. Studies associated with UA have been reported to possess anti-inflammatory properties, which are particularly relevant in liver cancer as chronic inflammation is a major risk factor for the development of HCC (Luan et al., 2022). A study has shown that UA significantly reduced the levels of inflammatory parameters IL-1β, IL-6 and TNF-α in mouse tissues. By reducing inflammation, UA helps to mitigate the progression of liver cancer (Zhao M. et al., 2023). Furthermore, UA has been shown to modulate various signaling pathways involved in liver cancer development and progression. A research study found that UA extracted from *Ludwigia hyssopifolia* can inhibit the activation of the PI3K/Akt/mTOR pathway, which is frequently dysregulated in liver cancer and promotes cell survival and proliferation (Liu et al., 2024). By targeting these signaling pathways, UA can exert its anti-cancer effects and inhibit the growth of liver tumors. Preclinical studies in H22 tumor-bearing mouse models have demonstrated the efficacy of UA in reducing tumor growth and improving survival outcomes (Wang et al., 2021). However, further research is required to further understand the therapeutic potential, evaluate the safety and efficacy of UA in liver cancer and optimize its use in clinical settings (Sun et al., 2020).

## 4 Function of ursolic acid on drug resistance and combination action

UA shows potential in combating drug resistance and boosting chemotherapy effectiveness in stomach cancer. Zhang et al. demonstrated that UA, when combined with oxaliplatin, effectively inhibited colorectal cancer (CRC) cell growth, increased cell death, and ROS production, thus preventing drug resistance (Zhang et al., 2018). Meng et al. found that UA boosted the anti-cancer effects of paclitaxel (PTX) in esophageal squamous cell carcinoma by inhibiting the Akt/FOXM1 cascade, leading to increased cell death and reduced cell mobility (Meng et al., 2021). Additionally, Zhao et al. showed that UA suppressed tumor growth by inhibiting the Wnt/β-catenin signaling system, thereby by slowing CRC growth, motility, clonality, and causes cell death (Zhao H. et al., 2023). Furthermore, Zhang et al. showed that UA was harmful to hepatoma cells that are resistant to multiple drugs, causing cell death through different pathways without changing P-glycoprotein expression (Zhang et al., 2007).

### 4.1 Role of nanotechnology and synergism with UA against gastrointestinal cancer

In the current scenario, NPs can be considered as a potential option for the treatment of various types of cancers that can accomplish various objectives limiting negative impact (Li et al., 2019). Nanoparticles can enhance drug delivery kinetics and the bio-distribution properties of medications (Ravindran et al., 2018). Novel NPs like nanobubbles have been created to enhance the accuracy and effectiveness of cancer diagnosis and treatment by delivering drugs to specific targets (Nittayacharn, et al., 2019).

### 4.2 Role of nanoparticles against gastrointestinal cancer

**FIGURE 5:**
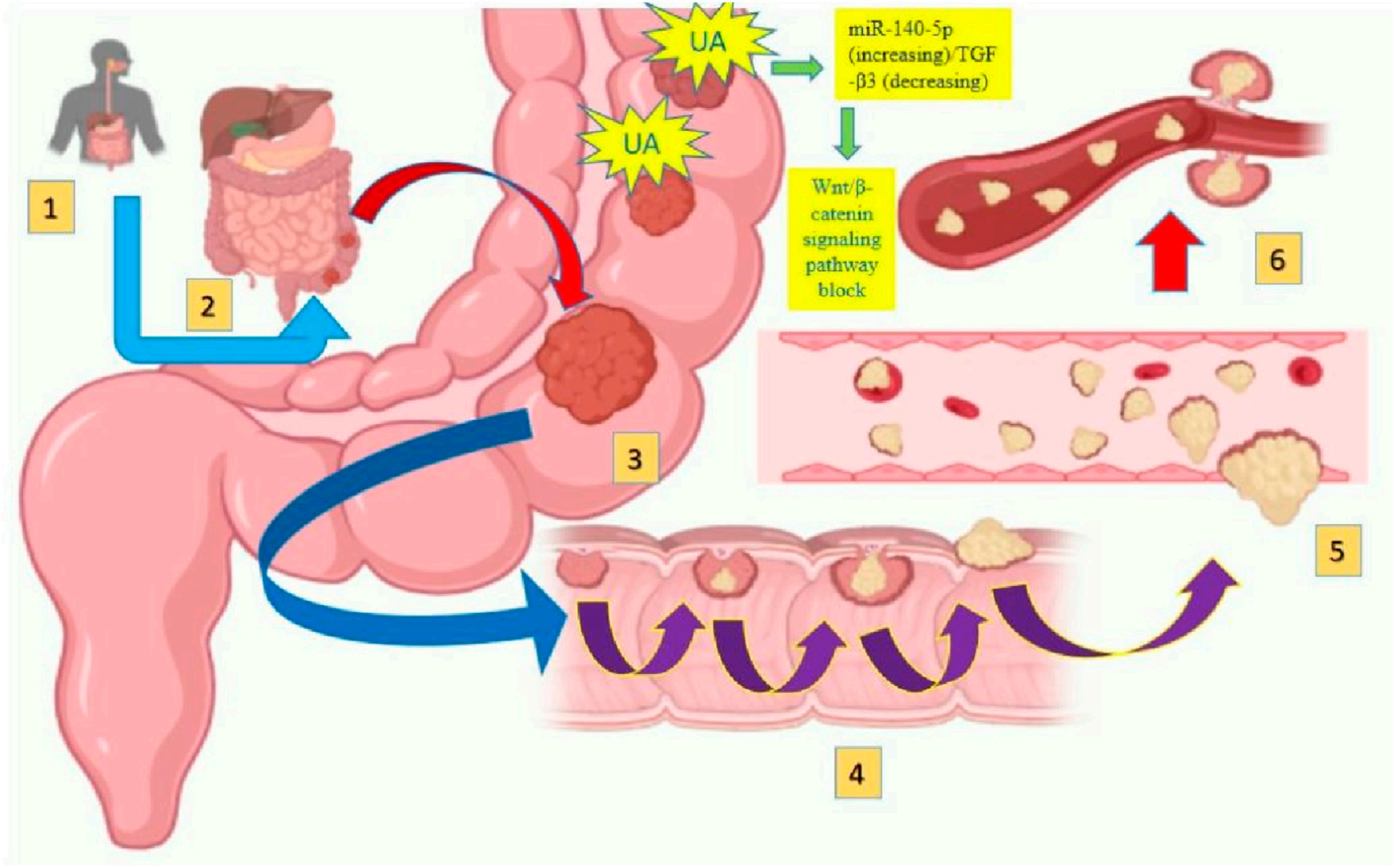
Developmental stages of colorectal cancer and inhibitory effect of ursolic acid.

Nanoparticles are essential in advancing the treatment of GI cancer. Nanoparticle-based research has shown promising results in targeting a wide range of cancer types due to its distinct characteristics (Kanaoujiya et al., 2022). Nanotechnology advancements, diagnosis and treatment are becoming more accessible. Throughout the years, a wide range of these particles have been utilized for diagnosing and treating cancers in the GI tract (Liang et al., 2022). Nanoparticle treatments offer numerous benefits in therapy for cancer, including their ability to carry a large amount of medication, pinpoint active tumors, and regulate drug release. Nanomaterials have been identified for potential use in treating gastric cancer shown in Figure 5 (Yao et al., 2020). Quantum dots have the potential to significantly contribute to the diagnosis of various cancer types through ongoing research on quantum dots probes (Khan et al., 2023). These are commonly used in identifying malignant tumors as a dependable sign. Examining the main components of the tumor stroma using a wide range of biomarkers to assess specific medical results in GI cancer (Wang H. et al., 2024).

In addition to quantum dots, dendrimers could also have a significant role. These are intricate branched artificial structures with multiple concentric layers. Various proteins can be identified through dendrimers. Furthermore, these can be utilized for imaging through Magnetic resonance imaging and Near-infrared spectroscopy modalities using a single probe (Fernandes et al., 2022). These NPs are designed with unique features tailored for use. Moreover, they can retain the drugs that fight cancer through encapsulation or chemical bonding with their surface functional groups (Joudeh and Linke, 2022).

**FIGURE 6:**
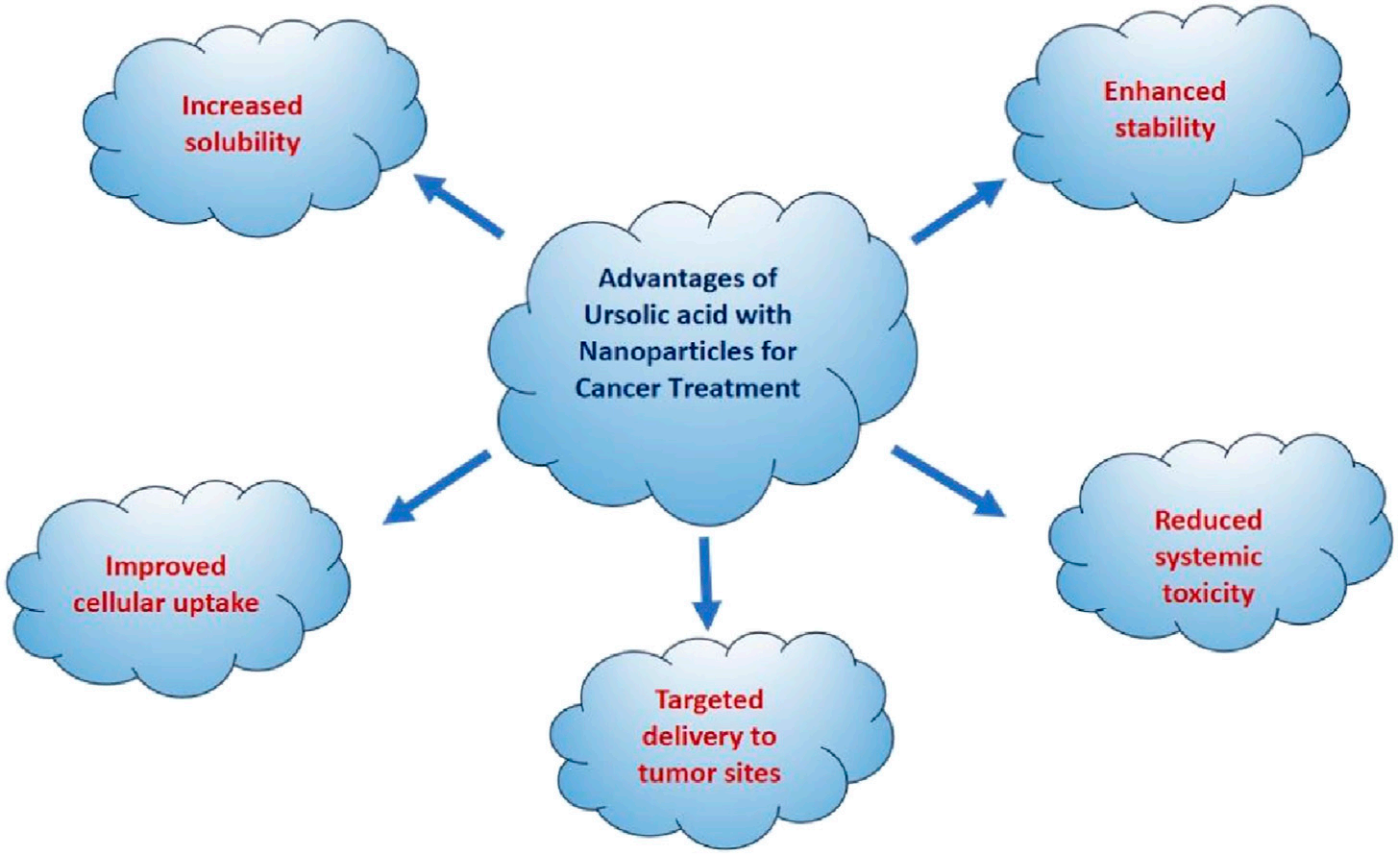
Beneficial effect of nanoparticles-based application in cancer therapy.

Moreover, when discussing certain metal NPs used for diagnosing and treating a particular disease, iron oxide NPs are considered excellent examples as they possess distinct magnetic responsiveness, surface functionalization, and biocompatibility. The surface of these NPs can be modified with specific ligands that can identify receptors over-expressed on the targeted cell for precise drug delivery (Figure 6) (Qiao et al., 2023). Alternatively, these NPs can be used for diagnosis and monitoring through imaging. The magnetic properties of these NPs enhance contrast for precise tumor detection. Credit to these distinct characteristics, they could be promising options for improving the effectiveness of treatments for GI tract cancer. In addition to these nanostructures, carbon nanotubes, nano-shells, nano-emulsions, liposomes, and polycaprolactone NPs are equally significant (Baranwal et al., 2023).

### 4.3 Synergistic effects of UA with nanoparticles

As far as GI mucosal permeability is concerned, UA has a low oral absorption rate and is poorly permeable. Hence, nanoformulations of UA are given intravenously to enhance drug delivery to the tumor. These formulations prioritize surface functional properties, stability, and size to enhance permeability and retention. Various nanoformulations have been researched over time, such as polymer micelles of UA, UA-liposomes, UA-nano-emulsions, UA-nanoparticles, Chitosan nanoparticles, polylactic acid nanoparticles, UA nanocrystals, etc. (Israel, 2018).

In this scenario, polymer micelles containing UA were created using mPEG-PLA (methoxy polyethylene glycol poly lactic acid) to target liver cancer cells. The delivery system displayed remarkable stability with a smooth and spherical shape, showcasing controlled release at various pH levels (7.5 and 5.5), leading to improved accumulation in tumors. This system inhibited HepG2 cell proliferation without harming normal hepatic cells and even enhanced normal hepatic cells at very low concentrations, indicating the potential of a UA-loaded polymer micellar delivery system for treating hepatic cancer (Zhou et al., 2019).

Enhanced stability with a slower release rate was noted in the Polyethylene glycol modified UA liposomes in comparison to regular liposomes. With a ratio of 3:2:5:50 UA, PEG, Cholesterol, and soy lecithin in PEG-modified UA liposomes, a uniformly spherical shape was achieved with a high encapsulation rate compared to regular liposomes. As a result, the liposome becomes harder, leading to enhanced membrane stability and preventing abrupt drug release (Zhao et al., 2015). Many nano-formulations have developed to enhance stability and improve the efficiency of drug release. Minimizing accumulation at non-targeted sites ultimately improves the clinical efficacy of UA. Liposomes could imitate cell membranes, enhancing the drug-delivery process (Lee et al., 2020). Furthermore, they struggle with temperature changes that can disrupt drug permeability and cause leakage. Just like micelles, there is a unique core-shell structure, but they have the lowest drug-loading capacity (Siboro et al., 2020). Nanocrystals exhibit high drug-loading capability and scalability, making them suitable for a wide range of applications (Jarvis et al., 2019; Naseema et al., 2021). In addition, nanoemulsions have unique properties and are highly responsive to environmental changes. Together, it is evident that each nano-formulation presents unique obstacles and benefits, with a focus on enhancing UA delivery methods for improved clinical results (Naseema et al., 2021).

## 5 Clinical safety aspects

Implementing the compound for clinical usage is the ultimate goal of all cancer research studies. Phase I trials are now being conducted on UA to assess its safety and potential side effects in patients. In the Biopharmaceutical Classification System (BCS), UA is categorized as a class IV drug with minimal pharmacological efficacy resulting from its poorly soluble nature in water and low permeability resulting in overall low bioavailability and effectiveness (Jinhua, 2019; Khwaza et al., 2020). To ascertain UA’s maximum tolerated dosage (MTD), pharmacokinetics (PK), and dose-limiting toxicities (DLT), 63 subjects—including healthy adults and individuals with advanced solid tumors were studied for UA administration as liposome (UAL). Each subject received one intravenous infusion of UAL (11, 22, 37, 56, 74, 98, and 130 mg/m^2^) during a period of 4 h. Clinical evidence demonstrated that UAL’s toxicity, with an MTD of 98 mg/m^2^, was manageable. DLTs included diarrhea and hepatotoxicity. UAL’s PK profile, however, was reported to be linear (Wang et al., 2013). Similarly, Zhu et al., 2013, examined the safety as well as single- and multiple-dose PK of UA nanoliposomes (UANL) in eight patients with advanced solid tumors and twenty-four healthy volunteers. The twenty-four healthy volunteers were split up into three groups and given a single dosage of UANL (37, 74, and 98 mg/m^2^) whereas eight individuals were administered with multiple UANL doses of 74 mg/m^2^ regularly for 14 days. Interestingly, for dose levels 37–98 mg/m^2^, the UANL was shown to be safe and to have an apparent linear PK pattern. Despite a 14-day continuous intravenous infusion, the repeated administration of UANL revealed no drug accumulation and was well tolerable in both patients and healthy volunteers. Another study investigated the multiple-dose safety and antitumor activity of UAL in advanced solid tumors subjects. UAL was injected intravenously into each individual for 14 consecutive days throughout a 21-day therapeutic cycle. To assess the efficacy and acceptability of multiple doses, twenty-one participants were enrolled in one of three consecutive cohorts (56, 74, and 98 mg/m^2^), additionally, eight subjects were investigated for multiple-dose PK with UAL (74 mg/m^2^). The results of a multiple-dose PK investigation indicated no accumulation of UAL in the body. Thus, UAL was identified as a tolerated drug with controllable toxicity that may increase the remission rates of patients (Qian et al., 2015). It’s evident from this research that UA holds great promise for becoming an effective anticancer medication.

## 6 Conclusion and future perspectives

Diverse pharmacological properties such as anti-proliferative, anti-inflammatory, and anti-metastatic activities make UA a potent therapeutic agent for GI cancers. However, poor permeability and low oral absorption pose challenges to the clinical use of this compound. Nano formulations such as polymer micelles and liposomes have been identified as potential solutions that improve UA delivery to tumors while enhancing drug stability. Phase I clinical trials on UA nano-formulations demonstrated tolerable toxicity profiles with linear pharmacokinetics indicative of their safety and efficacy profiles. In future, research should be aimed at optimizing UA nano-formulations so that they can be more useful in clinical settings. Additionally, novel strategies for treating GI cancers could result from the combination of UA with other treatment modalities like immunotherapy and targeted therapies. Molecular profiling in conjunction with nanoparticle-based drug delivery systems may open the door to individualized and successful treatment plans, which may ultimately improve the prognosis of patients with gastrointestinal cancers.

## References

[B1] Akşit H. Gözcü S. Altay A. (2023). Isolation and cytotoxic activities of undescribed iridoid and xanthone glycosides from *Centaurium erythraea* Rafn. (Gentianaceae). Phytochemistry 205, 113484. 10.1016/j.phytochem.2022.113484 36309110

[B2] Alam M. Ali S. Ahmed S. Elasbali A. M. Adnan M. Islam A. (2021). Therapeutic potential of ursolic acid in cancer and diabetic neuropathy diseases. Int. J. Mol. Sci. 22, 12162. 10.3390/ijms222212162 34830043 PMC8621142

[B3] Alsina M. Arrazubi V. Diez M. Tabernero J. (2023). Current developments in gastric cancer: from molecular profiling to treatment strategy. Nat. Rev. Gastroenterol. Hepatol. 20, 155–170. 10.1038/s41575-022-00703-w 36344677

[B4] Arnold M. Park J. Y. Camargo M. C. Lunet N. Forman D. Soerjomataram I. (2020). Is gastric cancer becoming a rare disease? A global assessment of predicted incidence trends to 2035. Gut 69, 823–829. 10.1136/gutjnl-2019-320234 32001553 PMC8520492

[B5] Baranwal J. Barse B. Di Petrillo A. Gatto G. Pilia L. Kumar A. (2023). Nanoparticles in cancer diagnosis and treatment. Materials 16, 5354. 10.3390/ma16155354 37570057 PMC10420054

[B6] Biller L. H. Schrag D. (2021). Diagnosis and treatment of metastatic colorectal cancer: a review. JAMA 325, 669–685. 10.1001/jama.2021.0106 33591350

[B7] Chan E. W. C. Soon C. Y. Tan J. B. L. Wong S. K. Hui Y. W. (2019). Ursolic acid: an overview on its cytotoxic activities against breast and colorectal cancer cells. J. Int. Med. 17, 155–160. 10.1016/j.joim.2019.03.003 30928277

[B8] Chen L. Liu M. Yang H. Ren S. Sun Q. Zhao H. (2022). Ursolic acid inhibits the activation of smoothened‐independent non‐canonical hedgehog pathway in colorectal cancer by suppressing AKT signaling cascade. Phytother. Res. 36, 3555–3570. 10.1002/ptr.7523 35708264

[B9] Chen Y. C. Malfertheiner P. Yu H. T. Kuo C. L. Chang Y. Y. Meng F. T. (2024). Global prevalence of *Helicobacter pylori* infection and incidence of gastric cancer between 1980 and 2022. Gastroenterology 166, 605–619. 10.1053/j.gastro.2023.12.022 38176660

[B10] Fan Y. Zhang X. Tong Y. Chen S. Liang J. (2022). Curcumin against gastrointestinal cancer: a review of the pharmacological mechanisms underlying its antitumor activity. Front. Pharmacol. 13, 990475. 10.3389/fphar.2022.990475 36120367 PMC9478803

[B11] Fernandes T. Daniel-da-Silva A. L. Trindade T. (2022). Metal-dendrimer hybrid nanomaterials for sensing applications. Coord. Chem. Rev. 460, 214483. 10.1016/j.ccr.2022.214483

[B12] González-Garibay A. S. López-Vázquez A. García-Bañuelos J. Sánchez-Enríquez S. Sandoval-Rodríguez A. S. Del Toro Arreola S. (2020). Effect of ursolic acid on insulin resistance and hyperinsulinemia in rats with diet-induced obesity: role of adipokines expression. J. Med. Food 23, 297–304. 10.1089/jmf.2019.0154 31747348

[B13] Guo J.-L. Han T. Bao L. Li X.-M. Ma J.-Q. Tang L.-P. (2019). Ursolic acid promotes the apoptosis of cervical cancer cells by regulating endoplasmic reticulum stress. J. Obstet. Gynaecol. Res. 45, 877–881. 10.1111/jog.13919 30632222

[B14] Hashem S. Ali T. A. Akhtar S. Nisar S. Sageena G. Ali S. (2022). Targeting cancer signaling pathways by natural products: exploring promising anti-cancer agents. Biomed. Pharmacother. 150, 113054. 10.1016/j.biopha.2022.113054 35658225

[B15] Hassanabad A. F. Chehade R. Breadner D. Raphael J. (2020). Esophageal carcinoma: towards targeted therapies. Cell. Oncol. (Dordr) 43, 195–209. 10.1007/s13402-019-00488-2 31848929 PMC12990719

[B16] Huang J. Fan X. Liu W. (2023). Applications and prospects of artificial intelligence-assisted endoscopic ultrasound in digestive system diseases. Diagnostics 13, 2815. 10.3390/diagnostics13172815 37685350 PMC10487217

[B17] Hui C. Ewongwo A. Lau B. Fisher G. Delitto D. Poultsides G. (2024). Patterns of recurrence after poor response to neoadjuvant chemotherapy in gastric cancer and the role for adjuvant radiation. Ann. Surg. Oncol. 31, 413–420. 10.1245/s10434-023-14350-1 37755563

[B18] Ikeda Y. Murakami A. Ohigashi H. (2008). Ursolic acid: an anti‐and pro‐inflammatory triterpenoid. Mol. Nutr. Food Res. 52, 26–42. 10.1002/mnfr.200700389 18203131

[B19] Israel L. L. (2018). A nanomedicine approach to manage cancer–imaging pancreatic cancer using targeted iron oxide nanoparticles. EBioMedicine 30, 7–8. 10.1016/j.ebiom.2018.03.011 29550240 PMC5952249

[B20] Jarvis M. Krishnan V. Mitragotri S. (2019). Nanocrystals: a perspective on translational research and clinical studies. Bioeng. Transl. Med. 4, 5–16. 10.1002/btm2.10122 30680314 PMC6336669

[B21] Jinhua W. (2019). Ursolic acid: pharmacokinetics process *in vitro* and *in vivo*, a mini review. Arch. Pharm. Weinh. 352, e1800222. 10.1002/ardp.201800222 30663087

[B22] Joudeh N. Linke D. (2022). Nanoparticle classification, physicochemical properties, characterization, and applications: a comprehensive review for biologists. J. Nanobiotechnol 20, 262. 10.1186/s12951-022-01477-8 PMC9171489 35672712

[B23] Kadasah S. F. Radwan M. O. (2023). Overview of ursolic acid potential for the treatment of metabolic disorders, autoimmune diseases, and cancers via nuclear receptor pathways. Biomedicines 11, 2845. 10.3390/biomedicines11102845 37893218 PMC10604592

[B24] Kalani K. Yadav D. K. Khan F. Srivastava S. K. Suri N. (2012). Pharmacophore, QSAR, and ADME based semisynthesis and *in vitro* evaluation of ursolic acid analogs for anticancer activity. J. Mol. Model. 18, 3389–3413. 10.1007/s00894-011-1327-6 22271093

[B25] Kanaoujiya R. Porwal D. Srivastava S. (2022). Applications of nanomaterials for gastrointestinal tumors: a review. Front. Med. Technol. 4, 997123. 10.3389/fmedt.2022.997123 36119898 PMC9475177

[B26] Kang D. Y. Sp N. Lee J.-M. Jang K.-J. (2021a). Antitumor effects of ursolic acid through mediating the inhibition of STAT3/PD-L1 signaling in non-small cell lung cancer cells. Biomedicines 9, 297. 10.3390/biomedicines9030297 33805840 PMC7998465

[B27] Kang D. Y. Sp N. Lee J.-M. Jang K.-J. (2021b). Antitumor effects of ursolic acid through mediating the inhibition of STAT3/PD-L1 signaling in non-small cell lung cancer cells. Biomedicines 9, 297. 10.3390/biomedicines9030297 33805840 PMC7998465

[B28] Khan M. S. Sheikh A. Abourehab M. A. Gupta N. Kesharwani P. (2023). Understanding the theranostic potential of quantum dots in cancer management. Mat. Today Comm. 36, 106424. 10.1016/j.mtcomm.2023.106424

[B29] Khwaza V. Oyedeji O. O. Aderibigbe B. A. (2020). Ursolic acid-based derivatives as potential anti-cancer agents: an update. Int. J. Mol. Sci. 21, 5920. 10.3390/ijms21165920 32824664 PMC7460570

[B30] Kim E. Moon A. (2015). Ursolic acid inhibits the invasive phenotype of SNU-484 human gastric cancer cells. Oncol. Lett. 9, 897–902. 10.3892/ol.2014.2735 25621065 PMC4301486

[B31] Kim K. Shin E. A. Jung J. H. Park J. E. Kim D. S. Shim B. S. (2018). Ursolic acid induces apoptosis in colorectal cancer cells partially via upregulation of MicroRNA-4500 and inhibition of JAK2/STAT3 phosphorylation. Int. J. Mol. Sci. 20, 114. 10.3390/ijms20010114 30597956 PMC6337206

[B32] Lagergren J. Smyth E. Cunningham D. Lagergren P. (2017). Oesophageal cancer. Lancet 390, 2383–2396. 10.1016/S0140-6736(17)31462-9 28648400

[B33] Lee N. R. Meng R. Y. Rah S. Y. Jin H. Ray N. Kim S. H. (2020). Reactive oxygen species-mediated autophagy by ursolic acid inhibits growth and metastasis of esophageal cancer cells. Int. J. Mol. Sci. 21, 9409. 10.3390/ijms21249409 33321911 PMC7764507

[B34] Li J. Liang X. Yang X. (2012). Ursolic acid inhibits growth and induces apoptosis in gemcitabine-resistant human pancreatic cancer via the JNK and PI3K/Akt/NF-κB pathways. Oncol. Rep. 28, 501–510. 10.3892/or.2012.1827 22641480

[B35] Li Z. Di C. Li S. Yang X. Nie G. (2019). Smart nanotherapeutic targeting of tumor vasculature. Accounts Chem Res 52, 2703–2712. 10.1021/acs.accounts.9b00283 31433171

[B36] Li Z.-Y. Chen S.-Y. Weng M.-H. Yen G.-C. (2021). Ursolic acid restores sensitivity to gemcitabine through the RAGE/NF-κB/MDR1 axis in pancreatic cancer cells and in a mouse xenograft model. J. Food Drug Anal. 29, 262–274. 10.38212/2224-6614.3346 35696208 PMC9261828

[B37] Liang M. Li L. D. Li L. Li S. (2022). Nanotechnology in diagnosis and therapy of gastrointestinal cancer. World J. Clin. Cases 10, 5146–5155. 10.12998/wjcc.v10.i16.5146 35812681 PMC9210884

[B38] Liang Y. Niu Q. Zhao Y. (2021). Pharmacological research progress of ursolic acid for the treatment of liver diseases. Trad. Med. Res. 6, 38. 10.53388/tmr20210331227

[B39] Limami Y. Pinon A. Wahnou H. Oudghiri M. Liagre B. Simon A. (2023). Ursolic acid’s alluring journey: one triterpenoid vs. Cancer hallmarks. Molecules 28, 7897. 10.3390/molecules28237897 38067626 PMC10707789

[B40] Lin C. W. Chin H. K. Lee S. L. Chiu C. F. Chung J. G. Lin Z. Y. (2019). Ursolic Acid induces apoptosis and autophagy in oral cancer cells. Environ. Toxicol. 34, 983–991. 10.1002/tox.22769 31062913

[B41] Lin J. Chen Y. Wei L. Shen A. Sferra T. J. Hong Z. (2013). Ursolic acid promotes colorectal cancer cell apoptosis and inhibits cell proliferation via modulation of multiple signaling pathways. Int. J. Oncol. 43, 1235–1243. 10.3892/ijo.2013.2040 23900560

[B42] Lin J.-H. Chen S.-Y. Lu C.-C. Lin J.-A. Yen G.-C. (2020). Ursolic acid promotes apoptosis, autophagy, and chemosensitivity in gemcitabine-resistant human pancreatic cancer cells. Phytother. Res. 34, 2053–2066. 10.1002/ptr.6669 32185829

[B43] Liu H. R. Ahmad N. Lv B. Li C. (2021). Advances in production and structural derivatization of the promising molecule ursolic acid. Biotechnol. J. 16, 2000657. 10.1002/biot.202000657 34096160

[B44] Liu T. Ma H. Shi W. Duan J. Wang Y. Zhang C. (2017). Inhibition of STAT3 signaling pathway by ursolic acid suppresses growth of hepatocellular carcinoma. Int. J. Oncol. 51, 555–562. 10.3892/ijo.2017.4035 28714512

[B45] Liu W. Kang S. Chen H. Bahetjan Y. Zhang J. Lu R. (2024). A composition of ursolic acid derivatives from Ludwigia hyssopifolia induces apoptosis in throat cancer cells via the Akt/mTOR and mitochondrial signaling pathways and by modulating endoplasmic reticulum stress. J. Ethnopharmacol. 319, 117351. 10.1016/j.jep.2023.117351 37884218

[B46] Liu X. Wang P. Zhang C. Ma Z. (2017). Epidermal growth factor receptor (EGFR): a rising star in the era of precision medicine of lung cancer. Oncotarget 8, 50209–50220. 10.18632/oncotarget.16854 28430586 PMC5564844

[B47] López-Hortas L. Pérez-Larrán P. González-Muñoz M. J. Falqué E. Domínguez H. (2018). Recent developments on the extraction and application of ursolic acid. A review. Food Res. Int. 103, 130–149. 10.1016/j.foodres.2017.10.028 29389599

[B48] Luan M. Wang H. Wang J. Zhang X. Zhao F. Liu Z. (2022). Advances in anti-inflammatory activity, mechanism and therapeutic application of ursolic acid. Mini Rev. Med. Chem. 22, 422–436. 10.2174/1389557521666210913113522 34517797

[B49] Ma X. Zhang M. Fang G. Cheng C. Wang M. Han Y. (2021). Ursolic Acid reduces hepatocellular apoptosis and alleviates alcohol-induced liver injury via irreversible inhibition of CASP3 *in vivo* . Acta Pharmacol. Sin. 42, 1101–1110. 10.1038/s41401-020-00534-y 33028983 PMC8209164

[B50] Meng R. Y. Jin H. Nguyen T. V. Chai O. H. Park B. H. Kim S. M. (2021). Ursolic acid accelerates paclitaxel-induced cell death in esophageal cancer cells by suppressing Akt/FOXM1 signaling cascade. Int. J. Mol. Sci. 22, 11486. 10.3390/ijms222111486 34768915 PMC8584129

[B51] Mlala S. Oyedeji A. O. Gondwe M. Oyedeji O. O. (2019). Ursolic Acid and its derivatives as bioactive agents. Molecules 24, 2751. 10.3390/molecules24152751 31362424 PMC6695944

[B52] Mu D. Zhou G. Li J. Su B. Guo H. (2018). Ursolic Acid activates the apoptosis of prostate cancer via ROCK/PTEN mediated mitochondrial translocation of cofilin-1. Oncol. Lett. 15, 3202–3206. 10.3892/ol.2017.7689 29435058 PMC5778871

[B53] Naseema A. Kovooru L. Behera A. K. Kumar K. P. Srivastava P. (2021). A critical review of synthesis procedures, applications and future potential of nanoemulsions. Adv. Colloid Interface Sci. 287, 102318. 10.1016/j.cis.2020.102318 33242713

[B54] Nittayacharn P. Yuan H. X. Hernandez C. Bielecki P. Zhou H. Exner A. A. (2019). Enhancing tumor drug distribution with ultrasound-triggered nanobubbles. J. Pharm. Sci. 108, 3091–3098. 10.1016/j.xphs.2019.05.004 31095958 PMC6708467

[B55] Panda S. S. Thangaraju M. Lokeshwar B. L. (2022). Ursolic Acid analogs as potential therapeutics for cancer. Molecules 27, 8981. 10.3390/molecules27248981 36558113 PMC9785537

[B56] Perestrelo R. Silva C. Fernandes M. X. Câmara J. S. (2019). Prediction of terpenoid toxicity based on a quantitative structure-activity relationship model. Foods 8, 628. 10.3390/foods8120628 31805724 PMC6963511

[B57] Pięt M. Paduch R. (2019). Ursolic and oleanolic acids as potential anticancer agents acting in the gastrointestinal tract. Mini Rev. Org. Chem. 16, 78–91. 10.2174/1570193x15666180612090816

[B58] Prasad S. Yadav V. R. Sung B. Gupta S. C. Tyagi A. K. Aggarwal B. B. (2016). Ursolic acid inhibits the growth of human pancreatic cancer and enhances the antitumor potential of gemcitabine in an orthotopic mouse model through suppression of the inflammatory microenvironment. Oncotarget 7, 13182–13196. 10.18632/oncotarget.7537 26909608 PMC4914350

[B59] Qian Z. Wang X. Song Z. Zhang H. Zhou S. Zhao J. (2015). A phase I trial to evaluate the multiple-dose safety and antitumor activity of ursolic acid liposomes in subjects with advanced solid tumors. Biomed. Res. Int. 2015, e809714. 10.1155/2015/809714 PMC4383362 25866811

[B60] Qiao R. Fu C. Forgham H. Javed I. Huang X. Zhu J. (2023). Magnetic iron oxide nanoparticles for brain imaging and drug delivery. Adv. Drug Deliv. Rev. 197, 114822. 10.1016/j.addr.2023.114822 37086918

[B61] Ravindran S. Suthar J. K. Rokade R. Deshpande P. Singh P. Pratinidhi A. (2018). Pharmacokinetics, metabolism, distribution and permeability of nanomedicine. Curr. Drug Metabol. 19, 327–334. 10.2174/1389200219666180305154119 29512450

[B62] Rawat L. Nayak V. (2021). Ursolic acid disturbs ROS homeostasis and regulates survival-associated gene expression to induce apoptosis in intestinal cancer cells. Toxicol. Res. 10, 369–375. 10.1093/toxres/tfab025 PMC8201588 34141150

[B63] Rawla P. Barsouk A. (2019). Epidemiology of gastric cancer: global trends, risk factors and prevention. Prz. Gastroenterol. 14, 26–38. 10.5114/pg.2018.80001 30944675 PMC6444111

[B64] Rustgi A. K. El-Serag H. B. (2014). Esophageal carcinoma. N. Engl. J. Med. 371, 2499–2509. 10.1056/NEJMra1314530 25539106

[B65] Sachdeo R. A. Charde M. S. Chakole R. D. (2020). Colorectal cancer: an overview. Asian J. Res. Pharm. Sci. 10, 211–223. 10.5958/2231-5659.2020.00040.5

[B66] Sawicki T. Ruszkowska M. Danielewicz A. Niedźwiedzka E. Arłukowicz T. Przybyłowicz K. E. (2021). A review of colorectal cancer in terms of epidemiology, risk factors, development, symptoms and diagnosis. Cancers (Basel) 13, 2025. 10.3390/cancers13092025 33922197 PMC8122718

[B67] Secrier M. Li X. De Silva N. Eldridge M. D. Contino G. Bornschein J. (2016). Mutational signatures in esophageal adenocarcinoma define etiologically distinct subgroups with therapeutic relevance. Nat. Gen. 48, 1131–1141. 10.1038/ng.3659 PMC5957269 27595477

[B68] Seely K. D. Morgan A. D. Hagenstein L. D. Florey G. M. Small J. M. (2022). Bacterial involvement in progression and metastasis of colorectal neoplasia. Cancers(Basel) 14, 1019. 10.3390/cancers14041019 35205767 PMC8870662

[B69] Shan J. Z. Xuan Y. Y. Zheng S. Dong Q. Zhang S. Z. (2009). Ursolic acid inhibits proliferation and induces apoptosis of HT-29 colon cancer cells by inhibiting the EGFR/MAPK pathway. J. Zhejiang Univ. Sci. B 10, 668–674. 10.1631/jzus.B0920149 19735099 PMC2738836

[B70] Shanmugam M. K. Manu K. A. Ong T. H. Ramachandran L. Surana R. Bist P. (2011). Inhibition of CXCR4/CXCL12 signaling axis by ursolic acid leads to suppression of metastasis in transgenic adenocarcinoma of mouse prostate model. Int. J. Cancer 129, 1552–1563. 10.1002/ijc.26120 21480220

[B71] Shapiro J. van Lanschot J. J. B. Hulshof M. C. C. M. van Hagen P. van Berge Henegouwen M. I. Wijnhoven B. P. L. (2015). Neoadjuvant chemoradiotherapy plus surgery versus surgery alone for oesophageal or junctional cancer (CROSS): long-term results of a randomised controlled trial. Lancet Oncol. 16, 1090–1098. 10.1016/S1470-2045(15)00040-6 26254683

[B72] Sheikh M. Roshandel G. McCormack V. Malekzadeh R. (2023). Current status and future prospects for esophageal cancer. Cancers (Basel) 15, 765. 10.3390/cancers15030765 36765722 PMC9913274

[B73] Shin J. Park Y. S. (2024). Unusual or uncommon histology of gastric cancer. J. Gastric Cancer 24, 69–88. 10.5230/jgc.2024.24.e7 38225767 PMC10774758

[B74] Siboro S. A. Salma S. A. Kim H. R. Jeong Y. T. Gal Y. S. Lim K. T. (2020). Diselenide Core cross-linked micelles of poly (ethylene oxide)-b-poly (Glycidyl methacrylate) prepared through alkyne-Azide click chemistry as a near-infrared controlled drug delivery system. Materials 13, 2846. 10.3390/ma13122846 32630421 PMC7344481

[B75] Somantri A. D. Kurnia D. Zainuddin A. Dharsono H. D. A. Satari M. H. (2021). Action mode of ursolic acid as a natural antioxidant and inhibitor of superoxide dismutase: *in vitro* and *in silico* study. J. Adv. Pharm. Technol. Res. 12, 389–394. 10.4103/japtr.japtr_90_21 34820315 PMC8588921

[B76] Song Y. Li L. Ou Y. Gao Z. Li E. Li X. (2014). Identification of genomic alterations in oesophageal squamous cell cancer. Nature 509, 91–95. 10.1038/nature13176 24670651

[B77] Souza M. Diaz I. Barchetta I. Mantovani A. (2024). Gastrointestinal cancers in lean individuals with non‐alcoholic fatty liver disease: a systematic review and meta‐analysis. Liver Int. 44, 6–14. 10.1111/liv.15763 37833849

[B78] Stitou M. Toufik H. Bouachrine M. Bih H. Lamchouri F. (2019). “Machine learning algorithms used in Quantitative structure-activity relationships studies as new approaches in drug discovery,” in 2019 *international Conference on intelligent Systems and advanced computing sciences* (ISACS) (Taza, Morocco: ISACS), 1–8.

[B79] Sun Q. He M. Zhang M. Zeng S. Chen L. Zhou L. (2020). Ursolic acid: a systematic review of its pharmacology, toxicity and rethink on its pharmacokinetics based on PK-PD model. Fitoterapia 147, 104735. 10.1016/j.fitote.2020.104735 33010369

[B80] Sung H. Ferlay J. Siegel R. L. Laversanne M. Soerjomataram I. Jemal A. (2021). Global cancer statistics 2020: GLOBOCAN estimates of incidence and mortality worldwide for 36 cancers in 185 countries. Cancer J. Clin. 71, 209–249. 10.3322/caac.21660 33538338

[B81] Sureda A. Martorell M. Capó X. Monserrat-Mesquida M. Quetglas-Llabrés M. M. Rasekhian M. (2021). Antitumor effects of triterpenes in hepatocellular carcinoma. Curr. Med. Chem. 28, 2465–2484. 10.2174/0929867327666200602132000 32484765

[B82] Thrift A. P. (2016). The epidemic of oesophageal carcinoma: where are we now? Cancer Epidemiol. 41, 88–95. 10.1016/j.canep.2016.01.013 26851752

[B83] Wang C. Gao Y. Zhang Z. Chen C. Chi Q. Xu K. (2020). Ursolic acid protects chondrocytes, exhibits anti-inflammatory properties via regulation of the NF-κB/NLRP3 inflammasome pathway and ameliorates osteoarthritis. Biomed. Pharmacother. 130, 110568. 10.1016/j.biopha.2020.110568 32745911

[B84] Wang H. Yang S. Chen L. Li Y. He P. Wang G. (2024). Tumor diagnosis using carbon-based quantum dots: detection based on the hallmarks of cancer. Bioact. Mater. 33, 174–222. 10.1016/j.bioactmat.2023.10.004 38034499 PMC10684566

[B85] Wang Z. Da Silva T. G. Jin K. Han X. Ranganathan P. Zhu X. (2014). Notch signaling drives stemness and tumorigenicity of esophageal adenocarcinoma. Cancer. Res. 21, 6364–6374. 10.1158/0008-5472.CAN-14-2051 PMC4527315 25164006

[B86] Wang L. Yin Q. Liu C. Tang Y. Sun C. Zhuang J. (2021). Nanoformulations of ursolic acid: a modern natural anticancer molecule. Front. Pharmacol. 12, 706121. 10.3389/fphar.2021.706121 34295253 PMC8289884

[B87] Wang S. Zheng R. Li J. Zeng H. Li L. Chen R. (2024). Global, regional, and national lifetime risks of developing and dying from gastrointestinal cancers in 185 countries: a population-based systematic analysis of GLOBOCAN. Lancet Gastroenterol Hepatol 9, 229–237. 10.1016/S2468-1253(23)00366-7 38185129 PMC10849975

[B88] Wang W.-J. Sui H. Qi C. Li Q. Zhang J. Wu S.-F. (2016). Ursolic acid inhibits proliferation and reverses drug resistance of ovarian cancer stem cells by downregulating ABCG2 through suppressing the expression of hypoxia-inducible factor-1α *in vitro* . Oncol. Rep. 36, 428–440. 10.3892/or.2016.4813 27221674

[B89] Wang X.-H. Zhou S.-Y. Qian Z.-Z. Zhang H.-L. Qiu L.-H. Song Z. (2013). Evaluation of toxicity and single-dose pharmacokinetics of intravenous ursolic acid liposomes in healthy adult volunteers and patients with advanced solid tumors. Expert Opin. Drug Metab. Toxicol. 9, 117–125. 10.1517/17425255.2013.738667 23134084

[B113] Weng H. Tan Z.-J. Hu Y.-P. Shu Y.-J. Bao R.-F. Jiang L. (2014). Ursolic acid induces cell cycle arrest and apoptosis of gallbladder carcinoma cells. Cancer Cell. Int. 1, 96. 10.1186/s12935-014-0096-6 PMC4224689 25383044

[B90] Woźniak Ł. Skąpska S. Marszałek K. (2015). Ursolic acid—a pentacyclic triterpenoid with a wide spectrum of pharmacological activities. Molecules 20, 20614–20641. 10.3390/molecules201119721 26610440 PMC6332387

[B91] Xi Y. Xu P. (2021). Global colorectal cancer burden in 2020 and projections to 2040. Transl. Oncol. 14, 101174. 10.1016/j.tranon.2021.101174 34243011 PMC8273208

[B92] Xiang F. Pan C. Kong Q. Wu R. Jiang J. Zhan Y. (2015). Ursolic Acid inhibits the proliferation of gastric cancer cells by targeting miR-133a. Oncol. Res. 22, 267–273. 10.3727/096504015X14410238486685 PMC7842598 26629938

[B93] Xie Y. H. Chen Y. X. Fang J. Y. (2020). Comprehensive review of targeted therapy for colorectal cancer. Signal Transduct. Target Ther. 5, 22. 10.1038/s41392-020-0116-z 32296018 PMC7082344

[B94] Yadav D. Mishra B. N. Khan F. (2018). 3D-QSAR and docking studies on ursolic acid derivatives for anticancer activity based on bladder cell line T24 targeting NF-kB pathway inhibition. J. Biomol. Struct. Dyn. 37, 3822–3837. 10.1080/07391102.2018.1528888 30261824

[B95] Yao Y. Zhou Y. Liu L. Xu Y. Chen Q. Wang Y. (2020). Nanoparticle-based drug delivery in cancer therapy and its role in overcoming drug resistance. Front. Mol. Biosci. 7, 193. 10.3389/fmolb.2020.00193 32974385 PMC7468194

[B97] Yin R. Li T. Tian J. X. Xi P. Liu R. H. (2018). Ursolic acid, a potential anticancer compound for breast cancer therapy. Crit. Rev. Food Sci. Nutr. 58, 568–574. 10.1080/10408398.2016.1203755 27469428

[B98] Yu D. Kan Z. Shan F. Zang J. Zhou J. (2020). Triple strategies to improve oral bioavailability by fabricating coamorphous forms of ursolic acid with piperine: enhancing water-solubility, permeability, and inhibiting cytochrome P450 isozymes. Mol. Pharm. 17, 4443–4462. 10.1021/acs.molpharmaceut.0c00443 32926628

[B99] Zafar S. Khan K. Hafeez A. Irfan M. Armaghan M. Rahman A. U. (2022). Ursolic acid: a natural modulator of signaling networks in different cancers. Cancer Cell. Int. 22, 399. 10.1186/s12935-022-02804-7 36496432 PMC9741527

[B100] Zhang D. M. Tang P. M. K. Chan J. Y. W. Lam H. M. Au S. W. N. Kong S. K. (2007). Anti-proliferative effect of ursolic acid on multidrug resistant hepatoma cells R-HepG2 by apoptosis induction. Cancer Biol. Ther. 6, 1377–1385. 10.4161/cbt.6.9.4528

[B101] Zhang N. Liu S. Shi S. Chen Y. Xu F. Wei X. (2020). Solubilization and delivery of Ursolic-acid for modulating tumor microenvironment and regulatory T cell activities in cancer immunotherapy. J. Control Release 320, 168–178. 10.1016/j.jconrel.2020.01.015 31926193

[B102] Zhang T. Xiang F. Li X. Chen Z. Wang J. Guo J. (2024). Mechanistic study on ursolic acid inhibiting the growth of colorectal cancer cells through the downregulation of TGF-β3 by miR-140-5p. J. Biochem. Mol. Toxicol. 38, e23581. 10.1002/jbt.23581 38044485

[B103] Zhang X. Li T. Gong E. S. Liu R. H. (2020). Antiproliferative activity of ursolic acid in MDA-MB-231 human breast cancer cells through Nrf2 pathway regulation. J. Agric. Food Chem. 68, 7404–7415. 10.1021/acs.jafc.0c03202 32551573

[B104] Zhang Y. Huang L. Shi H. Chen H. Tao J. Shen R. (2018). Ursolic acid enhances the therapeutic effects of oxaliplatin in colorectal cancer by inhibition of drug resistance. Cancer Sci. 109, 94–102. 10.1111/cas.13425 29034540 PMC5765292

[B105] Zhang Z. Wang J. Song N. Shi L. Du J. (2023). The global, regional, and national burden of stomach cancer among adolescents and young adults in 204 countries and territories, 1990–2019: a population-based study. Front. Public Health 11, 1079248. 10.3389/fpubh.2023.1079248 36908483 PMC9998989

[B106] Zhao H. Tang S. Tao Q. Ming T. Lei J. Liang Y. (2023). Ursolic acid suppresses colorectal cancer by down-regulation of Wnt/β-catenin signaling pathway activity. J. Agric. Food Chem. 71, 3981–3993. 10.1021/acs.jafc.2c06775 36826439

[B107] Zhao M. Wu F. Tang Z. Yang X. Liu Y. Wang F. (2023). Anti-inflammatory and antioxidant activity of ursolic acid: a systematic review and meta-analysis. Front. Pharmacol. 14, 1256946. 10.3389/fphar.2023.1256946 37841938 PMC10568483

[B108] Zhao T. Liu Y. Gao Z. Gao D. Li N. Bian Y. (2015). Self-assembly and cytotoxicity study of PEG-modified ursolic acid liposomes. Mater. Sci. Engg C 53, 196–203. 10.1016/j.msec.2015.04.022 26042707

[B109] Zhou M. Yi Y. Liu L. Lin Y. Li J. Ruan J. (2019). Polymeric micelles loading with ursolic acid enhancing anti-tumor effect on hepatocellular carcinoma. J. Cancer 10, 5820–5831. 10.7150/jca.30865 31737119 PMC6843872

[B110] Zhu Z. Qian Z. Yan Z. Zhao C. Wang H. Ying G. (2013). A phase I pharmacokinetic study of ursolic acid nanoliposomes in healthy volunteers and patients with advanced solid tumors. Int. J. Nanomed 8, 129–136. 10.2147/IJN.S38271 PMC3540956 23319864

[B111] Zou J. Lin J. Li C. Zhao R. Fan L. Yu J. (2019). Ursolic acid in cancer treatment and metastatic chemoprevention: from synthesized derivatives to nanoformulations in preclinical studies. Curr. Cancer Drug Targets 19, 245–256. 10.2174/1568009618666181016145940 30332961

[B112] Zou W. Luo X. Gao M. Yu C. Wan X. Yu S. (2024). Optimization of cancer immunotherapy on the basis of programmed death ligand‐1 distribution and function. Brit J. Pharmacol. 181, 257–272. 10.1111/bph.16054 36775813 PMC11080663

